# Gait Improvement by Alerted Push-Off via Heating of Insole Tip

**DOI:** 10.3390/healthcare10122461

**Published:** 2022-12-06

**Authors:** Kazushige Oshita

**Affiliations:** Department of Human Information Engineering, Okayama Prefectural University, 111 Kuboki, Soja, Okayama 719-1197, Japan; oshita@ss.oka-pu.ac.jp

**Keywords:** smart insole, stride length, walking, hip joint, leg swing, ankle plantar-flexion

## Abstract

This study investigated the change in the joint angles of the lower limb during gait by heating the tip of the insole to make a conscious push-off with the warm part. Fifteen healthy males performed treadmill walking under three different conditions: CONTROL walked as usual, INST was instructed to extend the stride with a push-off from the ball of foot to the toe, and HEAT was asked to walk while attempting to push off the warm area, which was attached to the disposable warmer to the area from the ball of foot to the toe of the insole. A 3D-motion capture system with infrared cameras was used to analyze the gait. The hip joint angle increased significantly under the INST and HEAT. Although the ankle dorsi-flexion at heel strike did not differ significantly for these conditions, ankle plantar-flexion significantly increased at toe-off under the INST and HEAT. Especially, effect size (*d*) in increased plantar-flexion was large in HEAT (=1.50), whereas it was moderate in INST (=0.68). These results suggest that a heated stimulus during gait enhanced the consciousness of push-off and increased leg swing and ankle plantar-flexion during the terminal stance phase, which may increase the stride length.

## 1. Introduction

Many so-called “smart insoles”, which provide users with information about their gait and running by embedding various sensors in the insole [1,2,3,4], have been developed in recent years and have attracted much attention [5]. Many of them measure the pressure or force applied to the sole [6] and display the trajectory of the center of foot pressure or estimate the stride length or cadence from it [5]. Most of the current smart insoles provide the information obtained by the insole to the user as visual information, so that users recognize their own gait (e.g., displaying the pressure distribution on a smartphone and providing data on stride length and number of steps as figures). When attempting to improve gait based on such information, users must intentionally change their own gait. However, a smart insole that analyzes information and stimulates the user to improve gait in real time, could become a more “advanced smart insole”.

In healthy older adults, more than half of falls are attributed to tripping during gait [7], and the minimum clearance, namely, the height between the plantar (toe) and floor during the swing phase of gait, is likely to cause tripping [8]. Guides or cues by some stimulus applied to the plantar region to increase the clearance in lower cadence individuals through the insole, could lead to the development of a smart insole that can prevent falls during gait. A study using tactile stimuli reported that when a small hemispherical protrusion was taped to the heel and the participants were instructed to step on the protrusion when the heel touches the floor, the ankle dorsi-flexion during heel strike and clearance during the swing phase increased [9]. This could also be applied to insoles; if a projection is attached to the heel of the insole and the individual attempts to land on the heel while stepping on a projectile, the insole will for prevent falls. This insole concept addresses the prevention of falls in the elderly. On the other hand, walking can be a purpose of exercise to increase physical activity and it is beneficial to increase physical activity through walking for all generations. This often focuses on the number of steps taken, and daily step goals are set to encourage increased physical activity. However, the amount of physical activity differs according to the magnitude of the stride length, even when the same number of steps is taken. Therefore, a smart insole that determines the decrease in stride length and a guiding or cueing stimulate the plantar to extend the stride length would help ensure physical activity.

Stride length and gait speed in healthy people have been found to decrease with age [10]. Shorter stride lengths have also been reported to be associated with lower muscle strength [11]. In particular, one of the hallmarks of age- or disability-related gait is a greater reduction in ankle power output during the final portion of the stance phase (terminal stance phase), and age-related impairment in ankle-power-generating capacity limits gait speed and step length [12]. In addition, with regard to the prevention of falls, the onset of diminished push-off in the terminal stance phase with aging may independently contribute to lower balance control and precipitate slower gait speeds [13]. These reports suggest that promoting appropriate push-off at the terminal stance phase and improving gait with sufficient stride length may be advantageous for the purpose of (1) exercise to increase physical activity, (2) preventing a decrease in gait speed with age, and (3) preventing falls from the perspective of balance control. Further, the previous study by Okoba at al. [9] revealed that when a small hemispherical protrusion was taped to the hallucal area, and the participants were instructed to walk placing the body weight on the protrusion seal during the kicking-out motion of the stride, increased plantar-flexion angle at terminal stance phase and increased plantar-flexor muscle (gastrocnemius) activity was observed.

**Figure 1:**
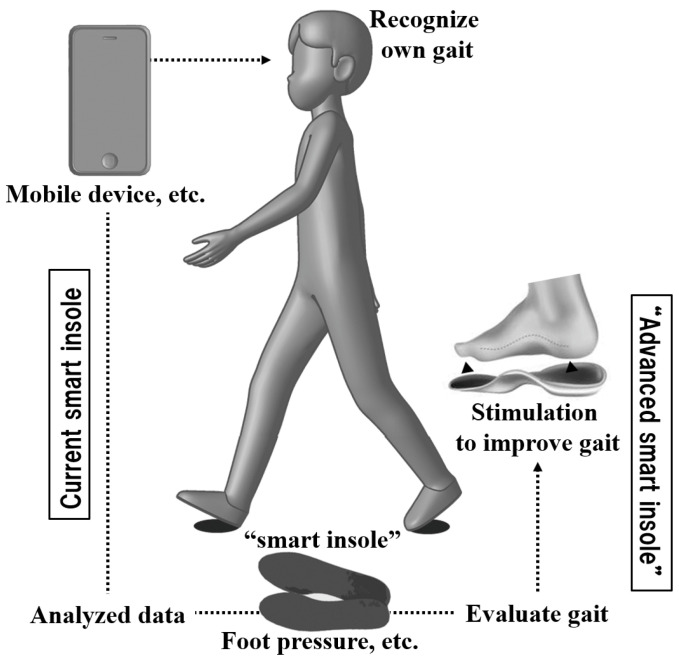
Schematic of the “advanced smart insole”.

Although tactile stimulation to the plantar may improve gait, it may be technically difficult to change the shape of the insole and create protrusions during walking. In contrast, heat-generating insoles with electrically heated elements embedded in the insole have been developed and marketed in recent years [14,15]. Most are charged by a power supply from USB or other sources and generate heat in response to the ambient temperature (e.g., in cold environments) to maintain a comfortable plantar temperature. The heating insole has a simple structure comprising an embedded heat-generating sheet and a small battery. If heat stimuli provided by the plantar, can increase the awareness of plantar sensation and alter foot movement during gait, advanced smart insoles, that can improve gait can be developed. In other words, the smart insole detects a decrease in stride length and heats the area from the ball of foot to the toe of the insole (Figure 1). Then, the stride length may increase if the user attempts to push off with the warm part. Similar gait improvement devices have been reported using eyeglasses [16] or a stimulator attached to the foot [17]. However, advanced smart insole could be used to improve gait with insoles alone. In order to develop such insoles, first of all, it is necessary to determine whether stimulation of the sole (tactile guiding or cueing) can actually lead the user to the target gait movement. Therefore, this study investigated the effect of heating the tip of the insole to facilitate conscious push-off with the warm part on the lower limb joint angles during gait.

## 2. Materials and Methods

### 2.1. Participants

The participants were 15 healthy males (age: 21–24 years) with no current or previous medical history of neural, muscular, or skeletal disorders. This study was conducted with younger participants, as the aim was to increase physical activity, i.e., exercise, by extending stride length. Prior to inclusion in the study, participants were informed of the purpose of the study, and informed consent was obtained from each participant. This study was approved by the ethics committee of the Okayama Prefectural University (No. 21-58).

The participants wore tight-fitting shirts and tights for motion capture, as described later. In addition, they wore shoes with a thin sole (5 mm thick) covered with an elastic cloth that fitted their feet perfectly (GA-5639, Kinugawa Co. Ltd., Tokyo, Japan) as insoles were used in this study. The shoes were available in various sizes, and the shoe that best fit the participant’s foot was selected.

### 2.2. Gait Task

**Figure 2:**
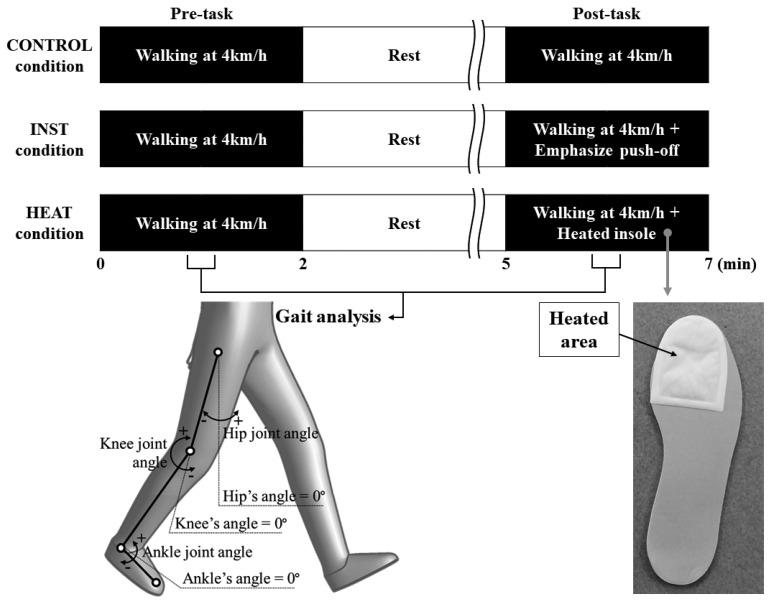
Experimental protocol and the definitions of each joint angle. (A disposable warmer was attached to the area from the ball of foot to the toe of the insole in post-task in HEAT condition. In the definitions of joint angles, positive values indicate flexion of hip and knee and ankle dorsiflexion. Further, negative values indicate extension of hip and knee and ankle plantarflexion).

Participants performed a 2 min walk at 4 km/h on a treadmill (Tempo T82, Johnson Health Tech, Taiwan) twice, with a 3 min break in between (The first and second gait tasks are referred to as “pre-“and “post-”, respectively) (Figure 2). This task was performed on three different days under the following three conditions. CONTROL condition: The participants walked as usual with a polyurethane insole in the shoe both pre- and post-task. INST condition: The pre-task was the same as in the control condition, walking normally; post-task, the participant was instructed to extend the stride with a push-off from the ball of foot to the toe. HEAT conditions: The pre-task was the same as in the control condition, walking normally; post-task, the insoles were replaced with modified ones during the break. A disposable warmer was attached to the area from the ball of foot to the toe of the insole and was heated (Figure 2). The temperature of the heated area, as measured using a thermometer (FS-300; HuBDIC Co., Ltd., Anyang-dong, Republic of Korea), was confirmed to be above 45 °C. The participants were instructed to walk while attempting to push off the warm area.

The order of the three conditions was randomized according to the participants. Participants unfamiliar with treadmill walking were given time to practice treadmill gait beforehand.

### 2.3. Motion Analysis

A 3D motion analysis system, consisting of infrared cameras (1728 × 1200 pixel) and motion capture software (MApro, ver. 1.0.1) (Hu-tech Co., Ltd., Tokyo, Japan) was used to collect kinematic data. Reflective markers were placed on the acromion, greater trochanter, lateral joint line of the knee, lateral malleolus, calcaneus, and third metatarsophalangeal joint. Six cameras were placed around the treadmill to capture all the reflective markers attached, and data were acquired at 240 fps during the middle 15 s of each gait task.

Acquired data were analyzed using 3D motion analysis software (ICpro-Analys, ver. 2.50, Hu-tech Co., Ltd., Tokyo, Japan) and smoothing using a second-order low-pass Butterworth filter with a cutoff frequency of 5 Hz was conducted. The definitions of each joint angle are shown in Figure 2. The hip joint angle was defined as the angle between the vertical line and the thigh (axis between greater trochanter and knee). The knee joint angle was defined as the angle between the thigh and the lower leg (axis between knee and lateral malleolus). The angle between the knee, lateral malleolus, and third metatarsophalangeal joint was defined as the ankle joint angle. These angular displacements were represented in percentage over the gait cycle course (0 to 100%). Further, the hip joint range of motion (ROM) was evaluated as the difference between the angle at initial contact and the angle at toe-off (i.e., the leg swing angle). The knee joint ROM was evaluated from the most flexed position to the most extended position, while the ankle joint ROM was evaluated from the most plantar-flexed position to the most dorsi-flexed position during a single step. The ankle joint angles at heel strike and toe-off were also evaluated. These angular variables were evaluated by calculating the average of four consecutive stable steps from the acquired data.

### 2.4. Statistical Analysis

A two-factor (pre- and post-task × three conditions) analysis of variance (ANOVA) was performed to compare the means for each angle and durations of one full gait cycle. Analyses results showed significance levels (*p*-values) and effect sizes (*η*^2^ and *f*-values). Further, post hoc analysis of achieved statical power (1-*β*) for observed significant effect in ANOVA was conducted using G*Power software (version 3.1.9.7) with *f*-value, α error, total sample size, number of groups and number of measurements. Multiple comparisons were performed using Holm’s method if a significant effect was observed. In addition to the significance tests, an effect size with Cohen’s *d* value was calculated for comparing the means of the pre- and post-tasks. ANOVA and multiple comparisons were performed using the js-STAR (ver. 9.8.6j, Japan) software. The level of statistical significance was set at <5%. The effect size was defined as small, moderate, or large depending on whether *d* < 0.2, 0.2 ≤ *d* < 0.8, or *d* ≥ 0.8, respectively.

## 3. Results

The durations of one full gait cycle in the CONTROL, INST, and HEAT conditions were 1.17 ± 0.07 s, 1.14 ± 0.08 s, and 1.16 ± 0.07 s for the pre-task, and 1.17 ± 0.08 s, 1.38 ± 0.10 s, and 1.38 ± 0.12 s for the post-task, respectively. Significant effects between pre-post (*p* < 0.01, *η*^2^ = 0.84, *f* = 2.31) and between conditions (*p* < 0.01, *η*^2^ = 0.66, *f* = 1.41) were observed, and their interaction was also significant (*p* < 0.01, *η*^2^ = 0.81, *f* = 2.06). The percentages of stance phase of the gait cycle on the CONTROL, INST, and HEAT conditions were 57.5 ± 4.2%, 57.8 ± 2.0%, and 58.6 ± 2.2% for the pre-task, and 58.1 ± 4.1%, 57.1 ± 2.5%, and 56.1 ± 5.0% for the post-task, respectively. No significant effects between pre-post and between conditions were observed, and their interaction was also not significant. These results indicate that although the duration of one gait cycle extended with instructions to push off with the toes or the tip of the insole heated and attempt to push off the warm part, the proportion of stance and swing phases was not significantly affected.

**Figure 3:**
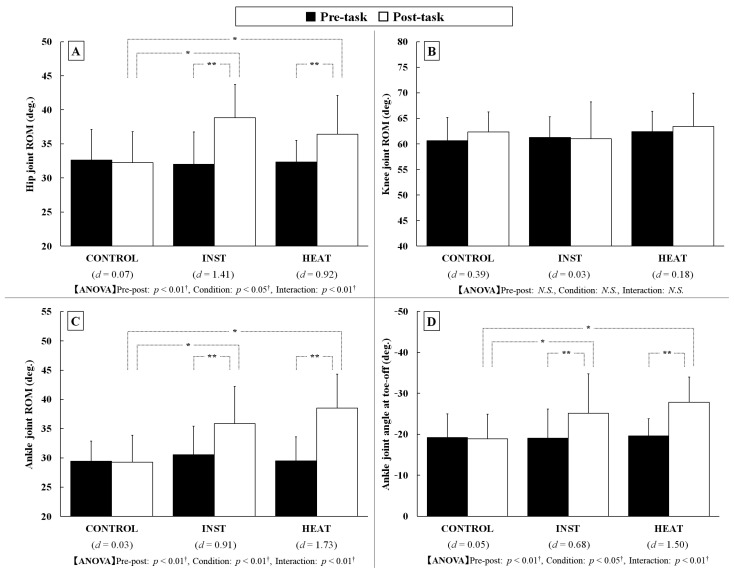
Hip (**A**), Knee (**B**), and ankle (**C**) joint ROM and ankle joint angle at heel-strike (**D**) during gait for the CONTROL, INST and HEAT condition (Values are means ± standard deviations. * and **: *p* < 0.05 and 0.01 (Holm’s multiple comparison). †: Statistical power (1 − *β*) ≥ 0.80).

**Figure 4:**
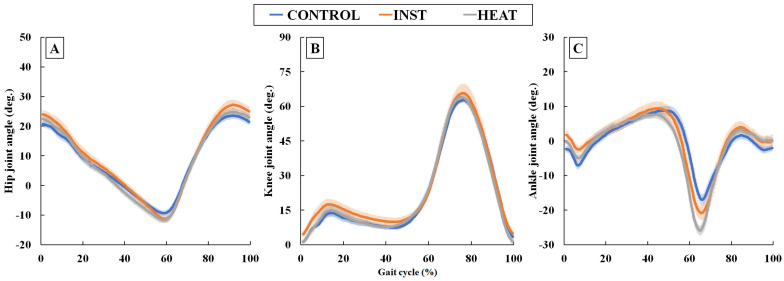
Changes in the angles of hip (**A**), knee (**B**), and ankle (**C**) movements in the sagittal plane during the gait cycle for the CONTROL, INST and HEAT condition in post-task (Values are means ± standard errors of mean). The definitions of each joint angle are shown in Figure 2.

The changes in the hip, knee, and ankle joint ROMs in each condition are shown in Figure 3A–C. The knee joint ROM exhibited no significant effects between the pre-post and between conditions whereas the hip joint ROM exhibited significant effects between the conditions (*p* < 0.05, *η*^2^ = 0.26, *f* = 0.60) and pre-post-task (*p* < 0.01, *η*^2^ = 0.64, *f* = 1.33), and their interaction was also significant (*p* < 0.01, *η*^2^ = 0.61, *f* = 1.25). Multiple comparisons in the hip angle ROM indicated significant increases in post-task compared to pre-task under the INST and HEAT conditions, with large effect sizes (*d* > 0.90). The ankle joint ROM exhibited significant effects between the conditions (*p* < 0.01, *η*^2^ = 0.49, *f* = 0.97) and pre-post (*p* < 0.01, *η*^2^ = 0.79, *f* = 1.92), and their interaction was also significant (*p* < 0.01, *η*^2^ = 0.41, *f* = 0.85). Multiple comparisons showed significant increases in post-task compared to pre-task in the INST and HEAT conditions, with large effect sizes (*d* > 0.90). Angular displacements in the sagittal plane of these three lower limb joints (hip, knee and ankle) for the CONTROL, INST and HEAT conditions in post-task are shown in Figure 4. These results indicate that the hip and ankle joint motion during gait increased with instructions to push off with the toes or the tip of the insole heated and attempt to push off the warm part.

The ankle joint angles at toe-off are shown in Figure 3D. The ankle joint angle at toe-off had significant effects between the conditions (*p* < 0.05, *η*^2^ = 0.20, *f* = 0.51) and pre-post (*p* < 0.01, *η*^2^ = 0.77, *f* = 1.81), and their interaction was also significant (*p* < 0.01, *η*^2^ = 0.42, *f* = 0.86). Multiple comparisons showed significant increases post-task compared to pre-task under the INST and HEAT conditions, with effect sizes that were moderate in the INST condition (*d* = 0.68) and large in the HEAT condition (*d* = 1.50). However, there were no significant effects of the ankle joint angle at heel strike in the pre-post and between conditions. The pre-post effect sizes for each condition were also less than moderate (*d* < 0.60). These results indicate that although instructions to push off with the toes or the tip of the insole heated and attempt to push off the warm part had no significant effect on the ankle joint angle at heel strike during gait, ankle plantar-flexion was increased at toe-off.

## 4. Discussion

This study investigated the changes in lower limb joint angles during gait by heating the ball of the foot to the toe of the insole and asking participants to extend their stride length to push-off with the warm part of the insole. Despite the limited sample size, this study provided the following results. The results indicated that although there was no significant effect on knee joint ROM under the experimental conditions, the hip joint ROM increased significantly under the INST and HEAT conditions. Although no significant effects were observed in the ankle joint angle at heel strike, the effects increased significantly at toe-off under the INST and HEAT conditions. Consequently, the ankle joint ROM increased significantly under the INST and HEAT.

Although the changes in ankle joint angle during one gait cycle seem to be almost the same between conditions, a difference appears to develop during the most plantar-flexed phase (Figure 4). Statistical analysis revealed that the ankle joint ROM was significantly larger in the post-task under the INST and HEAT condition in this study. Of these, ankle plantar-flexion at toe-off was considerably larger, while no significant difference occurred in the ankle joint angle at heel strike. Therefore, these results suggest that ankle plantar-flexion during the terminal stance phase was promoted by the attempt to push off the heated part of the insole (i.e., the area from the ball of the foot to the toe). Especially, effect size in increased ankle plantar-flexion at toe-off was large in HEAT condition (*d* = 1.50) whereas it was moderate in INST condition (*d* = 0.68). Therefore, thermal stimulation at the tip of the insole can make the participants aware of their specific toe-off position, which may lead to a more extended stride length. The importance of ankle plantar-flexion during the latter half of the stance phase, including the terminal stance phase, in accelerating or extending stride length during gait has been widely reported. For instance, the plantar-flexor muscles (soleus and gastrocnemius) combine to lift and accelerate the center of mass in the latter half of the stance phase of gait [18]. In particular, older adults possessed lower plantar-flexor power during the latter half of the stance phase of gait than young adults [19], even at fast speed [20]. Therefore, exercise training of the plantar-flexor muscles is suggested to be important for maintaining step length in advanced age [19]. These results suggest that the addition of stimulation by heating the tip of the insole during gait used in this study may be effective (1) in extending stride length and preventing future stride length shortening in young adults and (2) as a training approach to extend stride length in the elderly.

The present study showed that changes in knee joint angle during one gait cycle did not seem to differ among the three conditions (Figure 4), and statistical analysis showed no significant changes in knee joint ROM. Although changes in hip joint angle during gait cycle also appeared to be smaller differences among the three conditions (Figure 4), statistical analysis showed that not only ankle joint ROM but also hip joint ROM increased significantly in the INST and HEAT conditions, and these effect sizes were also large. The increase in hip joint ROM may be attributed to strong push-off during the terminal stance phase. Although stride length was not measured in this study because the participants walked on a treadmill, the duration of one gait cycle was increased in INST and HEAT condition. Increases in stride length are accompanied by both the aforementioned ankle plantar-flexion and activity in other lower limb muscle groups. A previous study showed that although increases in both the step length and cadence required larger contributions from the forces developed by the gluteus maximus, gluteus medius, vastus lateralis, gastrocnemius, and soleus for both vertical support and forward acceleration, an increase in step length resulted in greater differences in the contributions of the vastus lateralis, gluteus maximus, and limb posture to vertical support [18]. These results suggest that the addition of a heating stimulus at the tip of the insole during gait increases stride length, which may activate more lower limb muscle groups. It is, therefore, useful from the perspective of exercise training. However, extending stride length increases the single-leg support phase, which may lead to instability and an increased risk of falls in the elderly or individuals with poor balance capacity. Therefore, such a training approach should be aimed at preventing future decline in gait ability in individuals with sufficient physical fitness, and should not be utilized as a rehabilitation tool for those with poor physical fitness or gait impairments.

This study was conducted by simply wearing insoles with heated toes. Therefore, it is necessary to study cases where heating is added during walking. The final aim of the study was to apply a heating stimulus when the stride length decreased during walking. Furthermore, the temporary learning effect of thermal stimulation on gait improvement also needs to be investigated. Thermal stimulation of the soles should be temporary (only for a few minutes), because prolonged thermal stimulation may lead to discomfort in the shoes and may cause low-temperature burns. Assistance provided by light tactile cue to postural stability [21] and gait [22] has been reported to contribute to temporary these motor learning. Based on these studies, I speculate that that tactile cues provided by heated insoles may also be effective to improve gait, even if only temporarily. Therefore, studies are also needed to investigate whether the effect of temporary thermal stimulation of the plantar on stride lengthening remains even if the stimulation is removed afterwards. Currently, an experiment is conducting to investigate gait motion during and immediately after applying a temporary heating stimulus to sole by remotely activating a heater attached to the insole during long periods of walking. Furthermore, the health benefits of walking are reported to be greater at brisk/fast walking speeds than at the normal speeds [23] used in this study. Therefore, additional experiment is currently conducted faster walking speeds.

This study investigated the effects of heating the insole tip during gait on lower limb joint movements to develop an advanced smart insole, as described in the Introduction (Figure 1). The results demonstrated that such a heat stimulus increased ankle plantar-flexion during the terminal stance phase and the duration of one gait cycle, which may have led to an increase in stride length. However, this study has several limitations. First, the number of participants in this study was limited. Although 15 healthy males were included in this study, the number of participants needs to be increased and gender and/or age group should be taken into consideration. Considering the fact that females show a greater age-related decline in gait ability than males [24,25], female participants should be added to the study. Second, although this study was conducted on a treadmill for the purpose of motion analysis, investigation on level ground gait is also necessary. Finally, changes in muscle activity also need to be investigated as cited in the discussion. After addressing these issues and adding the results of the experiments currently underway, I would like to focus on developing of advanced smart insoles, i.e., to determine the decrease in stride length using the current smart insole technique, to heat the toe area at that time, and to verify the effect of this device.

## 5. Conclusions

This study investigated the change in the lower limb joint angles during walking by heating the tip of the insole to make a conscious push-off with the warm part. The results showed that although there was no significant effect between conditions on the knee joint ROM, the hip joint ROM increased significantly under the INST and HEAT conditions. Although no significant effects were observed in the ankle joint angle at heel strike, it significantly increased at toe-off under the INST and HEAT conditions. These results suggest that providing heat stimulus during gait enhances the consciousness of push-off and increases leg swing and ankle plantar-flexion during the terminal stance phase, which may increase the stride length. Especially, effect size in increased ankle plantar-flexion at toe-off phase was large in HEAT (*d* = 1.50) whereas it was moderate in INST (*d* = 0.68). Therefore, thermal stimulation at the tip of the insole can make the participants aware of their specific toe-off position, which may lead to a more extended stride length. Based on these results, future studies are expected to develop an advanced smart insole which is an insole device that determines the decrease in stride length using the current smart insole technique and heats the toe area at that time, and verify the effect of this device.

## Data Availability

The datasets generated and analyzed during the current study are available from the author on reasonable request.
